# The process of learning the autogenic training relaxation technique and its benefits on the wellness of people living with HIV

**DOI:** 10.1186/s12906-022-03557-6

**Published:** 2022-03-24

**Authors:** Maria Pilar Ramirez Garcia, Jérôme Leclerc-Loiselle, José Côté, Marie-Josée Brouillette, Réjean Thomas

**Affiliations:** 1 Faculty of Nursing, Université de Montréal, P. 6128, succ. Centre-Ville, Montréal, QC H3C 3J7 Canada; 2 Research Center of the Centre Hospitalier de l’Université de Montréal, P. 6128, succ. Centre-Ville, Montréal, QC H3C 3J7 Canada; 3 Quebec Nursing Intervention Research Network (RRISIQ), Quebec, QC Canada; 4 Department of Psychiatry, McGill University, Montréal, QC Canada; 5 AIDS and Infectious Disease Network (SIDA-MI), Quebec, Canada; 6 Clinique médicale l’Actuel, Montréal, Québec Canada

**Keywords:** Autogenic training, Mind-body, Wellness, Learning process, HIV

## Abstract

**Background:**

Various mind-body practices are used by people living with HIV to promote their general well-being. Among these is autogenic training (AT), a self-guided relaxation technique requiring regular practice for observable benefits. However, little has been written about the process of learning this technique, which is obviously a prerequisite to regular practice. This study therefore aims to describe the process by which people living with HIV learn AT.

**Methods:**

The study is a descriptive qualitative study using semi-structured interviews and a thematic analysis with a mixed approach. Fourteen participants living with HIV completed sessions to learn autogenic training over a period of 3 months.

**Results:**

The process of learning AT was approached through three themes: initiating the learning process, taking ownership of the technique, and observing its benefits on wellness. To initiate learning, participants had to express a need to take action on an aspect of their well-being and their openness to complementary approaches to care. Taking ownership of the technique was facilitated by guidance from the nurse researcher, the participants’ personal adaptations to overcome barriers to their practice, regular practice, and rapid observation of its benefits. Finally, the participants reported the observation of benefits on their wellness, including personal development, mainly in terms of the creative self, the essential self, and the coping self. This perception of the technique’s benefits was part of the learning process, as it contributed both to the participants’ ownership of the technique and to reinforcing their AT practice.

**Conclusions:**

People living with HIV see learning AT as a progressive process, in which wellness is a major outcome and a contributing factor in developing a regular practice.

**Supplementary Information:**

The online version contains supplementary material available at 10.1186/s12906-022-03557-6.

## Background

Improving the well-being of people living with human immunodeficiency virus (HIV) is a priority goal in HIV treatment. Indeed, the physical and emotional well-being of people living with HIV is lower than that of the general population, despite pharmacological advances [1]. The presence of various physical and psychological symptoms, such as sleep disorders, fatigue, muscle pain, anxiety, and symptoms of depression [2–11], may explain the lower level of well-being in this population.

People living with HIV report that HIV health professionals focus on management of physical health and do not address their broader goal of general well-being [12, 13]. To alleviate their symptoms, take a more active role in their health care and improve their sense of well-being, people living with HIV frequently use mind-body practices [13–15]. Mind-body practices focus on the connection between the mind, the body, and human behaviour to affect well-being. Provided or taught by a trained professional or well-qualified teacher, mind-body practices include yoga, tai-chi, acupuncture, meditation, massage therapy, and relaxation techniques [16].

Among these practices, autogenic training (AT), developed by Shultz, is a self-guided relaxation technique that aims to foster a state of calm and relaxation in the body and mind, by bringing attention to different bodily sensations [17]. This technique involves inducing a state of calm, performing a sequence of six standardized exercises [17–20], and finishing with a recovery exercise (Table 1) [17, 19]. It is recommended to practice AT three times a day, in sessions lasting between 5 and 15 min. The advantages of this technique over other mind-body practices are that it is easily learned and can be practiced independently from the very first day.

**Table 1:** Standardized Autogenic Training Exercises

Inducing a state of calm	
1. Heaviness	
2. Warmth	
3. Heartbeat	
4. Breathing	
5. Abdominal sensations	
6. Cool forehead	
Recovery: stretching, extending arms, inhaling, exhaling, and opening eyes	

AT has been shown to have positive effects on pain, anxiety, symptoms of depression, sleep disorders, fatigue, and quality of life [20–25] in various populations living with chronic disease. However, according to a systematic scoping review, few studies have evaluated the effects of AT on people living with HIV [26]. An experimental study with three groups—AT and progressive muscle relaxation (PMR) (*n* = 6), psychotherapy (*n* = 6), and usual care (*n* = 7)—showed a decrease in anxiety, depression, and fatigue in the first two groups after 12 weeks of practice [27]. Another pre-experimental study (*n* = 50) that combined AT with information on healthy lifestyle habits and cognitive-behavioural therapy showed effects on participants’ quality of life immediately after the sessions and 8 months later [28]. More recently, a mixed-method randomized feasibility study that compared AT (*n* = 14), PMR (*n* = 14), and usual care (*n* = 14) described participants’ perceived benefits of AT on quality of life, symptoms of depression, and emotion management [29]. Randomized controlled trials that evaluated the effects of AT in combination with other relaxation techniques and cognitive-behavioural therapy have shown positive effects on psychological symptoms and quality of life for people living with HIV [30–32]. However, these therapeutic interventions have multiple components, and are very complex and not easily accessible [26].

For the positive effects of AT on well-being to be observed, regular practice is essential [17, 29]. The repeated practice of AT increases the person’s ability to induce an ever-deeper state of relaxation and leads to the accumulation of therapeutic benefits [17]. Thus, greater understanding of the learning process that leads to regular AT practice would be relevant to supporting the learning process and improve well-being among people living with HIV. However, no studies appear to have focused on understanding the AT learning process as experienced by participants. Thus, this article aims to describe the process of learning the AT relaxation technique by people living with HIV.

This article is part of a randomized wait-list control trial, conducted to assess the effectiveness of an AT learning intervention on the quality of life and on the physical and psychological symptoms of people living with HIV. The AT learning intervention was implemented by the first author (PRG), who is a AT experimented and female nurse-researcher with more than 20 years of experience with this clientele. To learn AT, participants attended six, one-hour sessions over a three-month period and discuss their home-practice experiences with the nurse. The inclusion criteria for participation in this study were: 1) being over 18 years of age; 2) having been diagnosed with HIV; and 3) having or having had frequently in the past 2 weeks at least one of the following symptoms: sleep issues, fatigue, muscle pain, anxiety, or depression.

## Methods

A qualitative study with a descriptive estimate was conducted [33]. The study was approved by the Research Ethics Committee of the Centre hospitalier de l’Université de Montréal Research Centre (Reference: 15.013). Written informed consent was obtained from all participants prior to the randomization. Their initial consent included participation in the AT learning intervention, and in the qualitative research component after they learned the AT techniques. The Consolidated Criteria for Reporting Qualitative Research (COREQ) were used to guide presentation in the manuscript [34].

At the end of the six sessions, the first author (PRG) asked the participants in person whether they wished to participate in the qualitative research component. Therefore, convenience sampling was used. The only inclusion criterion was to have completed the AT-learning sessions. Participant recruitment was guided by data saturation [35], which was reached with the inclusion of 14 participants. Of these participants, 10 were in the initial intervention group and 4 were initially in the wait-list control group. The latter had to wait 6 months before learning AT. All participants asked agreed to participate, meaning no one refused.

The second author (JLL), who is a male PhD candidate, conducted semi-directed individual interviews by telephone between June 2016 and July 2018. JLL was not known to the participants and no characteristics were reported to participants prior to the interviews. Before each interview, JLL ascertained that consent had been given. One interview per participant was conducted on average 20 days after the end of the intervention. Lasting between 22 and 35 min, the interviews used open-ended questions to obtain the participants’ descriptions of the AT-learning process and the observed benefits for well-being. Considering our specific aim, interview questions focused on the process of learning AT, and this led to a shorter-than-usual duration for the qualitative interviews. An interview guide was developed by PRG and consisted of questions See Additional file 1 for more details. All the interviews were digitally recorded and transcribed.

A thematic analysis using a hybrid approach was performed [36]. The analysis was conducted using the concurrent steps developed by Miles, Huberman and Saldana [36], which are: 1) data condensation, 2) data presentation, and 3) development and validation of conclusions. For this purpose, PRG and JLL jointly developed an initial coding grid. This grid was deductively tested on the first four interviews, using blind parallel analysis with counter-coding. Open inductive coding was also performed. The coding grid was adjusted based on new categories after each interview was analyzed. The final coding grid had 83% inter-coder agreement. This grid was used to analyze the following 10 interviews. Excel software was used for data management and analysis.

The data were organized using a schematization process [36]. The purpose of these schemas was to describe the relationships between the code categories of the AT learning process and the perceived benefits on well-being. For the benefits, the development and verification of the conclusions were supported by the Indivisible Self wellness model [37].

This model [37] was used to interpret the results of this research following a finding in the inductive component that learning AT led to the development of general wellness. Myers, Sweeney and Witmer [38] defined wellness as: “a way of life orientated toward optimal health and well-being, in which body, mind, and spirit are integrated by the individual to live life more fully within the human and natural community” (p.252). Developed in individual psychology, this model uses five components to explain wellness: the coping self, which is made up of realistic beliefs, stress-management, self-worth, and leisure; the social self, which includes friendship and love; the creative self, which is comprised of thinking, emotions, control, positive humour, and work; the essential self, which includes spirituality and self-care; and the physical self that is comprised of exercise and nutrition. Myers, Sweeney and Witmer [38] put forth that mental health interventions, including mind-body practices like AT, enable the development of wellness via these five components. The benefits of AT practice have therefore been analyzed through an inductive thematization of these components. This wording was used deductively for the purpose of data organization.

The study’s rigour was ensured by adhering to the scientific criteria of reliability, credibility, confirmability, and transferability [39]. Two authors (PRG and JLL) independently coded four interview transcripts and compared their results to increase reliability and confirmability. The second author (JLL) coded the subsequent transcripts. Confirmability is also supported by the presentation of verbatim data. Although the transcripts were not returned to participants, reliability and credibility were also enhanced by the study’s iterative nature and the authors’ ongoing discussions of their interpretation of the results during analysis. In addition, a detailed description of the analytical framework, as well as the socio-demographic characteristics of the participants was carried out to enable readers to judge their transferability.

## Results

We conducted interviews with 14 participants (9 men and 5 women) with an average age of 54 years (37–65 years). All participants had been diagnosed with HIV between 28 and 420 months ago and had been on antiretroviral therapy for between 1 and 269 months. Eight participants self-identified as homosexual, five as heterosexual, and one as bisexual. Table 2 presents the participants’ socio-demographic characteristics.

**Table 2:** Participant Socio-demographics

Characteristics	Participants (*n* = 14)
Age (M ± SD)	54.36 ± 8.14
Sex	9 Male: 5 Female
HIV diagnosis (months)	223.64 ± 109.10
Antiretroviral therapy (months)	119.46 ± 102.96 (*n* = 13)
Marital status (*n* = 13)	8 single; 2 divorced; 2 married; 1 widowed
Sexual orientation	8 homosexual; 5 heterosexual; 1 bisexual

*M* Mean, *SD* Standard deviation

Interpretation of the data indicates that the AT learning process includes initiation to the technique, ownership of the technique, and the observed benefits of AT on wellness. The benefits of this practice on the various dimensions of wellness appear gradually with practice and also contribute to taking ownership of the AT practice. First, we will present the central elements of the AT learning process, including its benefits for wellness. Second, we will explain the place of wellness development in the learning process. Table 3 depicts the categories of analysis.

**Table 3:** List of Analysis Codes and Categories

Purpose of the study	Analysis categories	Themes	
**Process of learning AT**	Initiation to AT	Needing to take action on an aspect of their well-being	
		Openness to complementary approaches	
	Ownership of AT	Guidance from the nurse	
		Personal adaptations to overcome barriers to practice	Environment
			Daily living
			Exercises
		Regular practice	
		Rapid observation of benefits	
	The benefits of AT practice on wellness	The coping self	Ability to handle stressful situations
			Resumption of certain leisure activities
			Awareness of personal capabilities
		The social self	Development of social relationships
		The creative self	Ability to regulate thoughts and emotions
			Openness to positive things and events
			Perception of control
		The essential self	Self-care exercises
		The physical self	Resumption of physical exercise

*AT* Autogenic training

### From initiation to benefits for wellness

The process of learning AT was approached through three categories: 1) initiation to AT, 2) ownership of AT, and 3) the benefits of AT practice on wellness. For data presentation, each category encompasses themes that are underlined. Some themes integrate sub-themes, which are shown in italics. Table 3 clarifies the relationship between categories, themes, and sub-themes.

#### 1. Initiation to AT

Before asking participants to learn the AT technique, we sought two necessary prerequisites in their profile: a need to take action for their well-being and an openness to complementary approaches.

##### Needing to take action on an aspect of their well-being

All the participants expressed a need to take action on some aspect of their well-being that called for immediate attention. This could be a global need, such as taking a break from everyday life or a strategy for coping with recurrent issues, or it could be a need related to a symptom, such as sleeping issues, depression, or anxiety. In all cases, participants needed tools and strategies that would offer immediate results.

*“I think that when I started with [PRG], I was very depressed and I really needed a technique that would help me relax and recentre, to support me”* (60-64 years-old female).

##### Openness to complementary approaches

All participants were receptive to complementary approaches. This openness may have developed as a result of previous experimentation with other mind-body practices, such as yoga and PMR, or with other complementary practices, such as eye movement desensitization and reprocessing. It may also stem from a desire to try an approach that is complementary to modern medicine and pharmacotherapy.

“*For me it was easy. It was really easy because I had already started doing meditation. So it was not hard to relax*.” (55-59 years-old male).

#### 2. Ownership of AT

Taking ownership of the AT practice seems to be a central factor in the learning process and to developing a sense of mastery, which in turn is key to maintaining a regular practice. This appropriation was facilitated by: guidance from the nurse, the participants’ personal adaptations to overcome barriers to their practice, regular practice, and rapid observation of its benefits.

##### Guidance from the nurse

The guidance of a nurse trained in the AT relaxation technique and the development of a trust-based relationship are considered necessary to participants learning the AT technique. Our participants reported that the nurse offered a safe and supportive guiding presence and kept them focused. The nurse also led discussions about their learning, including possible adaptations to overcome barriers to practice.

*“Just [the nurse] saying: ‘I’m calm, I feel calm’ reassured me. It helped me see that there was no danger”* (45-49 years-old male).

##### Personal adaptations to overcome barriers

Participants were required to make certain personal adaptations to overcome perceived barriers to their AT practice. These hurdles had to do with the environment, daily life, and certain AT exercises.

*Environment* Some participants perceived the physical environment as an impediment to practice. While the room chosen by the nurse was seen as an ideal place to learn the technique, because it was quiet, calm, and dark, the participants noted that their home practice was often interrupted by noise, phone calls, and the presence of family members. This made their daily practice difficult to sustain at first. Participants reported that, over time, they were able to shut out the noise, but that they had to take steps to help themselves concentrate at the beginning of the learning process. These measures included secluding themselves or reserving time when they knew their environment would be more conducive to concentrating.

*“I don’t know how. I don’t know if it’s because the doors are closed, but I feel in my own bubble and it’s easier for me to practice in my car.”* (50–54 years-old male)

*Daily life* The participants perceived daily life to be a major obstacle, and found it difficult to fit the AT practice into an established routine. Participants mentioned that especially work- or family-related stress kept them from taking time to do their AT practice. To get over this hurdle, participants either adopted a discipline-based approach, by taking a break from their tasks at specific predetermined times, or a flexibility-based one, by reducing the number of sessions each day. However, a key element of the analysis was to ensure regular daily practice.

*“It was a little harder; three times a day was too much. In the end, I now practice very regularly once a day. […] I don’t feel like this hampered the benefits.”* (55-59 years-old female).

*Autogenic training exercises* Some of the AT exercises were particularly difficult to integrate for the participants. This was especially so for exercises that asked participants to feel a sensation of warmth or their heartbeat. The participants preferred to adapt these exercises, mostly by word substitution, to better reflect their perception.

*“I never felt the warmth. So instead of warmth, I said gentleness or calm. Because there was some […], I said: ‘I feel calm.’”* (65–69 years-old male)

##### Regular practice

A necessary condition for learning AT was building a regular practice into the participants’ daily routine by setting times for their AT practice. These times were mostly linked to meals or to certain activities, like waking up or going to bed. Once routine, this regular practice promoted learning, the observation of benefits, and the gradual ownership of the AT practice. Thus, regular practice progressively became easier and more automatic.

*“It’s an interesting technique, and relatively easy to practice. The only thing is in the beginning, it needs to be done regularly. You got to think about it every time. But at some point, it becomes automatic.”* (35–39 years-old male)

##### Rapid observation of benefits

Participants had to quickly observe the benefits of their practice on their wellness. Several perceived a sense of wellness from the very first practice onward. This motivated them to keep up their learning and make personal adaptations. Conversely, the participants who did not feel the technique’s effect on the problematic symptomatology said that this made them think about dropping out during the first few days. However, with practice, the participants gradually observed greater and longer-lasting beneficial effects on their symptoms and overall wellness, and this seemed to motivate them to continue their practice.

*“At first, it didn’t work. My body wouldn’t learn. Maybe it was the stress or something… It was hard to let go. But when you get the upper hand, suddenly everything falls into place. […] It is not always perfect with stress, and work. But it got me involved.”* (60–64 years-old male)

#### 3. The benefits of AT practice on wellness

The benefits of AT practice on wellness were analyzed through the five components described by Myers and Sweeney [37] We will present them successively:

##### The coping self

In this component, learning AT promoted individuals’ ability to manage stressful situations, to resume leisure activities, and to become aware of their personal capabilities.

Some participants mentioned that learning AT helped them develop their ability to manage stressful situations, both personal and professional. AT became a tool that enabled them to take a step back from such situations, which facilitated more thoughtful decision-making. They reported feeling calmer as a result of their AT practice.

*“[Before], I use methods that had so-so results. [With AT, however], it was easy for me just to stop working and take a break for a few minutes”* (50-54 years-old male)*.*

Some participants mentioned regaining interest in certain leisure activities, like reading, cooking, or watching television, that they had stopped due to fatigue. They explained that AT gave them energy to engage in activities they wanted to do.

A last major element was the participants’ awareness of their personal capabilities, especially in facing the challenges of daily life. Learning AT was associated with a process of personal development that enabled them to adapt to life situations. Some participants began seeing certain situations less as insurmountable impediments and more as hurdles they could overcome. After learning AT, participants reported being able to get through the day more easily.

*“It doesn’t change the problem, but it takes the edge off. AT helps me cope with what’s going on in my life”* (65-69 years-old male).

##### The social self

Some participants mentioned that their social relationships had improved as a result of their AT practice. The participants were more interested in spending time with their loved ones and had more energy to engage in social activities. Some participants mentioned that their friends and families had commented on them being calmer and more pleasant to be around. The participants felt more satisfied with their social relationships.

*“Other people [have told me]: ‘you’re calmer, you’re less angry’ I’ve received a lot of compliments about that”* (40-44 years-old female)*.*

##### The creative self

The creative self is the component that emerges most strongly from the analyses, on three different levels. The participants reported an improvement in their ability to regulate their thoughts and emotions, a perception of greater self-control, and a greater openness to the positive things in life.

With learning AT, the participants spoke of an increase in their ability to regulate their thoughts and emotions. Several participants mentioned that their thoughts prevented them from concentrating at the beginning of their practice. In a majority of cases, AT reduced the flow of thoughts and helped participants redirect, relativize, and even eliminate dark thoughts. Some participants also mentioned that learning AT had allowed them to develop better emotional regulation. They reported having fewer fluctuations and therefore a more stable emotional state.

*“I would say concentration [is the main effect], in terms of my thoughts. When you stop thinking, you’re at rest. For me, I think that’s the big difference”* (50-54 years-old male).

Participants reported observing a sense of self-control, including an ability to concentrate and calm down. They also experienced for the first time an effect on their physical symptoms, such as tremors or pain, over which they previously had no control.

*“[AT] helped me tame my tremors and overcome my fear. When I’m practicing AT, I am almost tremor-free.”* (50–54 years-old female)

Finally, some participants also mentioned an increase in their openness to positive things and events in their lives. Some participants noted that the sense of control gained through their AT practice made them more inclined to make time for things that were good for them.

##### The essential self

Some participants saw AT as an exercise in self-care for better living and mental health. Participants mentioned that AT was gradually becoming a need, allowing them to get through their daily lives better. AT influenced many facets of their lives and its impact reached well beyond their sessions. Indeed, it would seem that their practice provided them with a sense of general wellness that improved their overall quality of life. AT fit into a healthy lifestyle and the participants’ desire to improve their lives.

*“It makes me aware of life, of the things around me. I take time to do things: I cook, I visit museums, I go to the park”* (55-59 years-old male)*.*

##### The physical self

Finally, on this level, a few participants mentioned that the introduction of AT had motivated them to do more physical exercise. Although less common in the interviews, a few participants mentioned that this technique led them to want to improve other aspects of their lives, and that they did so by joining sports centres or doing outdoor activities.

*“[I’m] more motivated about getting exercise. I feel like doing it more than I used to. Before, I was too tired, like ‘ah no, not that!’ Now, I’ve got more drive”* (55–59-year-old male)*.*

### From wellness to ownership of the AT technique and strengthening practice

As mentioned above, participants began learning AT after identifying a need to take action to improve an aspect of their wellness that, to maintain the learning process, needed to be addressed quickly. The participants mentioned that observing benefits of their practice was key to their desire to continue learning and practicing AT. Indeed, participants made the AT technique their own and reinforced a regular practice by progressively observing the benefits to their wellness. Their perception of wellness was therefore part of the learning process as a result of their AT practice, as well as a contributing factor to taking ownership of and consolidating a regular AT practice.

**Fig. 1:**
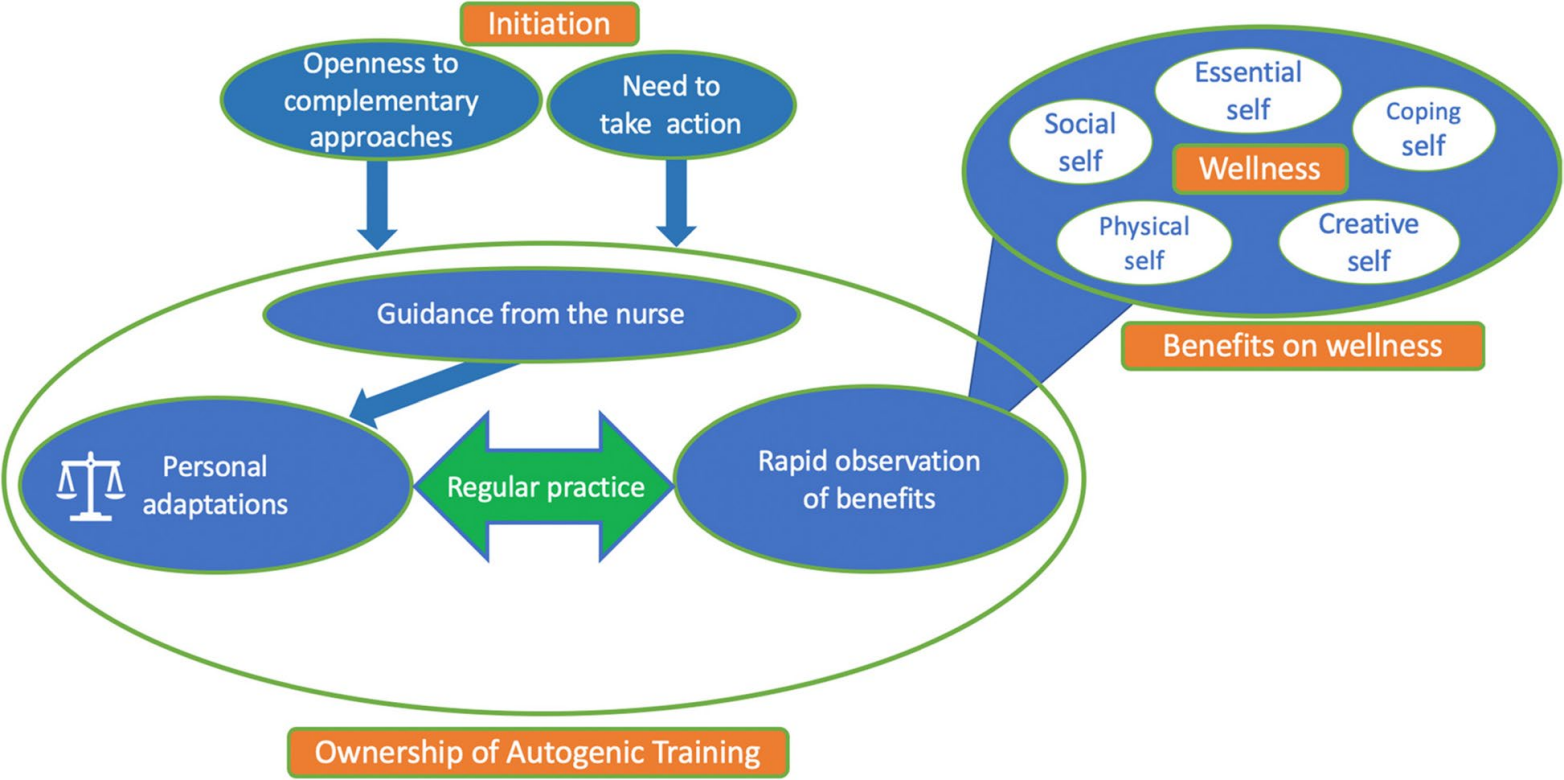
Process of Learning Autogenic Training

In Fig. 1, we illustrate the learning process that takes place between taking ownership of AT and the benefits of wellness for people living with HIV.

*“It makes me feel that wellness. So it is not just [an intervention] to cure me of anxiety. […] It’s becoming a new need”* (55-59-year-old male)*.*

## Discussion

Our study shows that the process of learning the AT relaxation technique begins with a person’s willingness to act on some aspect of their well-being, and it seems to be built on the participants’ previous experiences of other mind-body practices, nurse support, personal adaptations, regular practice, and observation of the technique’s benefits, particularly on wellness.

The participants in our study observed the positive effects of AT practice on their wellness, as conceived by Myers and Sweeney [37]. Many participants reported feeling more self-confident, allowing themselves to take more time for them, being able to resume activities they had stopped, and taking a more positive outlook on life. These results are consistent with those of a study proposing a model for the psychological mechanism of AT effects [40]. Participants had reported reduced anxiety and improved mood and well-being, specifically perceiving regular AT practice to contribute to better thought and emotional management: they felt like a different person, who was happier or more optimistic. This study describes the awareness of body sensations as the core of the AT experience [40].

While there is limited empirical evidence explaining the mechanism behind those effects of AT, such findings have been well established for mindfulness-based interventions. An emotion-regulation model called Mindfulness-to-Meaning Theory (MAT) [41–43] has been put forward to explain the cognitive and emotional mechanisms of mindfulness. According to MAT, mindfulness evokes a metacognitive process of consciousness that transforms our experience, promotes positive re-evaluation, and facilitates positive emotions and coping behaviours [41–43]. It would seem that positive re-evaluation leads to increased self-awareness, a focus on finding the silver lining, and the ability to put a positive spin on a situation, which in turn all enhance positive emotions and increase wellness [41–43]. For these authors, mindfulness is a natural psychological capacity [41] that involves bringing and sustaining attention to one object without cognitive or emotional attachment [44]. This capacity can be increased with mindfulness-based interventions [41], but also by an everyday activity, performed with intentionality and awareness [44]. As of yet, there is no empirical evidence as to the effectiveness of AT in increasing mindfulness. However, as practiced in our study, AT involves repeatedly drawing attention and awareness to various body sensations. Our results suggest that AT could indeed increase mindfulness and that the effects AT participants perceived on their wellness could be explained by the MAT mechanism.

Furthermore, Peper, Harvey, and Lin [45] suggest that mindfulness-based interventions share similar strategies with AT and other mind-body practices, such as paying attention, shifting intention, and observing and letting go of thoughts, emotions and external events. Considering very few studies have examined AT participants’ learning experiences, the comparison between our results and those of other studies, including on mind-body and mindfulness practices, will provide a better understanding of the mechanisms at play.

The results of our study also support Schultz’s [17] assertion that *willpower* is a necessary condition for a person to start this technique. With respect to nurse support, a study on anxiety described participants’ acknowledgement of the counsellor’s guidance in learning the AT technique and sharing their personal experiences [40]. The importance of the instructor’s role in supporting participants and adapting exercises was also reported in a study that described the experience of participating in a yoga program for people with diabetes [46].

In our study, participants identified stress and environmental or daily life factors as interfering with their AT practice. A study to better understand mindfulness meditation training by participants living with chronic insomnia [47] identified that time, daily routine, and environment can be barriers to practice. Other studies, on yoga among people living with diabetes [46] and mindfulness meditation among adolescents living with chronic pain and other somatic symptoms [48], also identified lack of time as a barrier to practice. However, participants in the latter study reported that early symptom relief overcame this barrier and facilitated engagement in the practice [48]. In addition, the importance of regular practice was recognized as a process that was essential to participants achieving results in most mind-body practices [49]. In this sense, personal adaptations to circumvent obstacles to learning and practice, such as those related to the environment and daily life, seem to be key considerations when teaching AT. We believe that using strategies to teach AT that are based on theoretical foundations and empirical literature on habit development [50–53], such as intention planning [50, 54, 55] and the repetition of behaviour in the same context-situation [56, 57], may have contributed to these adaptations and to participants integrating the practice into their daily routines.

## Conclusion

In sum, the process of learning AT involves initiating learning, taking ownership of the technique, and observing its benefits for wellness. This learning seems to be facilitated by nurse guidance, which helps participants make personal adaptations to overcome the obstacles to regular practice and to observe benefits quickly. The benefits of AT practice on participants’ wellness—which extend beyond physical and psychological symptoms—also contribute to them taking ownership of the technique and their development of a regular practice. Our findings thus highlight the progressive nature of the learning process, with the development of wellness as a major outcome and contributing factor in participants developing a regular practice.

## Supplementary Information

**Additional file 1.** Interview guide for AT participants.

## Data Availability

The datasets generated and/or analysed during the current study are not publicly available due to risks of identifying participants but are available from the corresponding author on reasonable request.

## Abbreviations

AT: Autogenic Training
COREQ: Consolidated Criteria for Reporting Qualitative Research
HIV: Human Immunodeficiency Virus
M: Mean
MAT: Mindfulness-to-Meaning Theory
PMR: Progressive Muscle Relaxation
SD: Standard deviation

## References

[1] Hutton VE, Misajon R, Collins FE (2013). Subjective wellbeing and ‘felt’ stigma when living with HIV. Qual Life Res.

[2] Briongos Figuero LS, Bachiller Luque P, Palacios Martin T, Gonzalez Sagrado M, Eiros Bouza JM (2011). Assessment of factors influencing health-related quality of life in HIV-infected patients. HIV Med.

[3] Harding R, Clucas C, Lampe FC, Date HL, Fisher M, Johnson M, Edwards S, Anderson J, Sherr L (2012). What factors are associated with patient self-reported health status among HIV outpatients? A multi-centre UK study of biomedical and psychosocial factors. AIDS Care.

[4] Lalanne C, Armstrong AR, Herrmann S, Le Coeur S, Carrieri P, Chassany O, Duracinsky M (2015). Psychometric assessment of health-related quality of life and symptom experience in HIV patients treated with antiretroviral therapy. Qual Life Res.

[5] Herrmann S, McKinnon E, Hyland NB, Lalanne C, Mallal S, Nolan D, Chassany O, Duracinsky M (2013). HIV-related stigma and physical symptoms have a persistent influence on health-related quality of life in Australians with HIV infection. Health Qual Life Outcomes.

[6] Gamaldo CE, Spira AP, Hock RS, Salas RE, McArthur JC, David PM, Mbeo G, Smith MT (2013). Sleep, function and HIV: a multi-method assessment. AIDS Behav.

[7] Zimpel RR, Fleck MP (2014). Depression as a major impact on the quality of life of HIV-positive Brazilians. Psychol Health Med.

[8] Pompili M, Pennica A, Serafini G, Battuello M, Innamorati M, Teti E, Girardi N, Amore M, Lamis DA, Aceti A (2013). Depression and affective temperaments are associated with poor health-related quality of life in patients with HIV infection. J Psychiatr Pract.

[9] Namisango E, Harding R, Atuhaire L, Ddungu H, Katabira E, Muwanika FR, Powell RA (2012). Pain among ambulatory HIV/AIDS patients: multicenter study of prevalence, intensity, associated factors, and effect. J Pain.

[10] Parker R, Stein DJ, Jelsma J (2014). Pain in people living with HIV/AIDS: a systematic review. J Int AIDS Soc.

[11] George S, Bergin C, Clarke S, Courtney G, Codd MB (2016). Health-related quality of life and associated factors in people with HIV: an Irish cohort study. Health Qual Life Outcomes.

[12] Bristowe K, Clift P, James R, Josh J, Platt M, Whetham J, Nixon E, Post F, McQuillan K, Ní Cheallaigh C (2019). Towards person-centred care for people living with HIV: what core outcomes matter, and how might we assess them? A cross-national multi-Centre qualitative study with key stakeholders. HIV Med.

[13] Littlewood RA, Vanable PA (2011). A global perspective on complementary and alternative medicine use among people living with HIV/AIDS in the era of antiretroviral treatment. Curr HIV/AIDS Rep.

[14] Lorenc A, Robinson N (2013). A review of the use of complementary and alternative medicine and HIV: issues for patient care. AIDS Patient Care STDs.

[15] Abou-Rizk J, Alameddine M, Naja F (2016). Prevalence and characteristics of CAM use among people living with HIV and AIDS in Lebanon: implications for patient care. Evid Based Complement Alternat Med.

[16] Mind and body practices. [https://www.nccih.nih.gov/health/mind-and-body-practices]. Accessed 21 Dec 2021.

[17] Schultz JH (1958). Le training autogène.

[18] Kanji N, White AR, Ernst E (2006). Autogenic training for tension type headaches: a systematic review of controlled trials. Complement Ther Med.

[19] Luthe W, Peper E, Ancoli S, Quinn M (1979). About the Methods of Autogenic Therapy. Mind/Body Integration: Essential Readings in Biofeedback.

[20] Stetter F, Kupper S (2002). Autogenic training: a meta-analysis of clinical outcome studies. Appl Psychophysiol Biofeedback.

[21] Kanji N, White AR, Ernst E (2004). Autogenic training reduces anxiety after coronary angioplasty: a randomized clinical trial. Am Heart J.

[22] Wright S, Courtney U, Crowther D (2002). A quantitative and qualitative pilot study of the perceived benefits of autogenic training for a group of people with cancer. Eur J Cancer Care (Engl).

[23] Sutherland G, Andersen MB, Morris T (2005). Relaxation and health-related quality of life in multiple sclerosis: the example of autogenic training. J Behav Med.

[24] Asbury EA, Kanji N, Ernst E, Barbir M, Collins P (2009). Autogenic training to manage symptomology in women with chest pain and normal coronary arteries. Menopause.

[25] Bowden A, Lorenc A, Robinson N (2012). Autogenic training as a behavioural approach to insomnia: a prospective cohort study. Prim Health Care Res Dev.

[26] Ramirez-Garcia MP, Gagnon M-P, Colson S, Côté J, Flores-Aranda J, Dupont M (2019). Mind-body practices for people living with HIV: a systematic scoping review. BMC Complement Altern Med.

[27] Fukunishi I, Hosaka T, Matsumoto T, Hayashi M, Negishi M, Moriya H (1997). Liaison psychiatry and HIV infection (II): application of relaxation in HIV positive patients. Psychiatry Clin Neurosci.

[28] Kermani KS (1987). Stress, emotions, autogenic training and AIDS: a holistic approach to the management of HIV-infected individuals. Holistic Med.

[29] Ramirez-Garcia MP, Leclerc-Loiselle J, Gagnon MP, Côté J, Brouillette MJ, Thomas R (2020). A mixed-method randomized feasibility trial evaluating progressive muscle relaxation or autogenic training on depressive symptoms and quality of life in people living with human immunodeficiency virus (HIV) who have depressive symptoms. J Complement Integr Med.

[30] McCain NL, Munjas BA, Munro CL, Elswick RK, Robins JLW, Ferreira-Gonzalez A, Baliko B, Kaplowitz LG, Fisher EJ, Garrett CT (2003). Effects of stress management on PNI-based outcomes in persons with HIV disease. Res Nurs Health.

[31] Cruess S, Antoni MH, Hayes A, Penedo F, Ironson G, Fletcher MA, Lutgendorf S, Schneiderman N (2002). Changes in mood and depressive symptoms and related change processes during cognitive–behavioral stress management in HIV-infected men. Cognit Ther Res.

[32] Antoni MH, Pereira DB, Marion I, Ennis N, Andrasik MP, Rose R, McCalla J, Simon T, Fletcher MA, Lucci J (2008). Stress management effects on perceived stress and cervical neoplasia in low-income HIV-infected women. J Psychosom Res.

[33] Tesch R. Qualitative research: analysis types and software: London: Taylor & Francis; 2013.

[34] Tong A, Sainsbury P, Craig J (2007). Consolidated criteria for reporting qualitative research (COREQ): a 32-item checklist for interviews and focus groups. Int J Qual Health Care.

[35] Morse JM (2015). Data were saturated…. Qual Health Res.

[36] Miles MB, Huberman AM, Saldana J (2014). Qualitative data analysis.

[37] Myers JE, Sweeney TJ (2004). The indivisible self: an evidence-based model of wellness. J Individ Psychol.

[38] Myers JE, Sweeney TJ, Witmer JM (2000). The wheel of wellness counseling for wellness: a holistic model for treatment planning. J Couns Dev.

[39] Lincoln YS, Guba EG (1985). Naturalistic inquiry, vol. 9.

[40] Yurdakul L, Holttum S, Bowden A (2009). Perceived changes associated with autogenic training for anxiety: a grounded theory study. Psychol Psychother.

[41] Garland EL, Hanley AW, Goldin PR, Gross JJ (2017). Testing the mindfulness-to-meaning theory: evidence for mindful positive emotion regulation from a reanalysis of longitudinal data. PLoS One.

[42] Garland EL, Thielking P, Thomas EA, Coombs M, White S, Lombardi J, Beck A (2017). Linking dispositional mindfulness and positive psychological processes in cancer survivorship: a multivariate path analytic test of the mindfulness-to-meaning theory. Psychooncology.

[43] Garland EL, Farb NA, Goldin P, Fredrickson BL (2015). Mindfulness broadens awareness and builds eudaimonic meaning: a process model of mindful positive emotion regulation. Psychol Inq.

[44] Hanley AW, Warner AR, Dehili VM, Canto AI, Garland EL (2015). Washing dishes to wash the dishes: brief instruction in an informal mindfulness practice. Mindfulness.

[45] Peper E, Harvey R, Lin I-M (2019). Mindfulness training has elements common to other techniques. Biofeedback.

[46] Thind H, Guthrie KM, Horowitz S, Conrad M, Bock BC (2019). "I can do almost anything": The experience of adults with type 2 diabetes with a yoga intervention. Complement Ther Clin Pract.

[47] Hubbling A, Reilly-Spong M, Kreitzer MJ, Gross CR (2014). How mindfulness changed my sleep: focus groups with chronic insomnia patients. BMC Complement Altern Med.

[48] Ali A, Weiss TR, Dutton A, McKee D, Jones KD, Kashikar-Zuck S, Silverman WK, Shapiro ED (2017). Mindfulness-based stress reduction for adolescents with functional somatic syndromes: a pilot cohort study. J Pediatr.

[49] Cairns V, Murray C (2015). How do the features of mindfulness-based cognitive therapy contribute to positive therapeutic change? A meta-synthesis of qualitative studies. Behav Cogn Psychother.

[50] Lally P, Wardle J, Gardner B (2011). Experiences of habit formation: a qualitative study. Psychol Health Med.

[51] Neal DT, Wood W, Quinn JM (2006). Habits—a repeat performance. Curr Dir Psychol Sci.

[52] Nilsen P, Roback K, Broström A, Ellström PE (2012). Creatures of habit: accounting for the role of habit in implementation research on clinical behaviour change. Implement Sci.

[53] Wood W, Neal DT (2007). A new look at habits and the habit-goal interface. Psychol Rev.

[54] Lally P, Chipperfield A, Wardle J (2008). Healthy habits: efficacy of simple advice on weight control based on a habit-formation model. Int J Obes.

[55] Orbell S, Verplanken B (2010). The automatic component of habit in health behavior: habit as cue-contingent automaticity. Health Psychol.

[56] Lally P, van Jaarsveld CHM, Potts HWW, Wardle J (2010). How are habits formed: modelling habit formation in the real world. Eur J Soc Psychol.

[57] Verplanken B (2006). Beyond frequency: habit as mental construct. Br J Soc Psychol.

