# Supplementary material for: The process of learning the autogenic training relaxation technique and its benefits on the wellness of people living with HIV
Source: BMC Complement Med Ther. 2022 Mar 24;22:86. doi: 10.1186/s12906-022-03557-6 (PMC8953141; doi:10.1186/s12906-022-03557-6)
Supplement: Supplementary file 1 — Additional file 1. Interview guide for AT participants. [file 12906_2022_3557_MOESM1_ESM.docx]

**Additional file 1**

**Interview guide for AT participants**

The original guide was developed in French. A translation is proposed here.

General questions about the process experienced by the participants

1. For the past three months you have been participating in an intervention to learn autogenic training and to develop the habit of practicing this relaxation technique daily at home.

a. Can you tell me about the experience of **learning** AT?

b. What did you learn from this intervention?

2. During the sessions, the nurse asked you to practice this method daily at home as well as to note your sensations and how you felt after the practice.

a. Can you tell me about your experience of practicing AT at home?

b. What was easier for you to do?

c. What was harder for you to do?

General questions on participants' perceptions of the effects of AT

3. We would now like to know what you have experienced from the relaxation method that you have now been practicing for three months.

a. Can you tell me about how you were feeling and the symptoms you had before you started the intervention? What about now?

b. Can you tell me about the quality of life you had before you started the intervention? What about now?

c. Can you tell us what you did when you had symptoms like fatigue, pain, sleeping problems, anxiety or depressive symptoms before the intervention? What are you doing now?

d. In your opinion, what are the impacts of this intervention?

Conclusion

4. For three months, you have been learning AT. We want to eventually be able to use it in other settings with other people living with HIV.

a. As a participant, do you have anything to add or advice to give us to help us improve this intervention?
